# Survival and Prognostic Factors in Unresectable Head and Neck Cancer Patients

**DOI:** 10.3390/jcm14155517

**Published:** 2025-08-05

**Authors:** Natsuki Oishi, Sara Orozco-Núñez, José Ramón Alba-García, Mar Gimeno-Coret, Enrique Zapater

**Affiliations:** 1 ENT Department, General University Hospital of Valencia, 46014 Valencia, Spain; saonuez@alumni.uv.es (S.O.-N.); alba_josgar@gva.es (J.R.A.-G.); gicomar@uv.es (M.G.-C.);; 2 Faculty of Medicine and Odontology, Valencia University, 46010 Valencia, Spain

**Keywords:** head and neck cancer, unresectable, overall survival, prognostic factors

## Abstract

**Background/Objectives:** This single-cohort follow-up study describes the median overall survival (OS) in patients with unresectable head and neck squamous cell carcinoma (HNSCC) due to invasion of vital structures, which is under-represented in the current literature. Secondarily, subgroups were evaluated according to the type of presentation, in order to identify clinical characteristics and contribute to developing an appropriate treatment plan and managing patient’s expectations. **Methods:** This single-cohort observational study analysed the OS of 39 patients from the Otolaryngology Department with advanced-stage head and neck cancer with invasion of vital anatomical structures considered ineligible for surgical treatment. Secondarily, subgroups were evaluated according to type of presentation and various clinical characteristics. **Results:** A total of 39 patients radiologically classified as having unresectable HNSCC (i.e., unsuitable for surgical resection), with a mean age of 66.87 years, were included during a 24-month follow-up. By the end of the study, 56.4% of the patients had died. The median OS was 16.09 months. Statistically significant differences were observed when comparing human papilloma virus (HPV)-positive and -negative status and when comparing initial and recurrent tumours. **Conclusions:** The invasion of anatomical structures such as the skull base, internal carotid artery, and prevertebral space was associated with a marked decrease in survival, with an OS time of 16 months. This study provides valuable evidence in patients with unresectable HNSCC, highlighting tumour recurrence and HPV-negative status as important indicators of poor prognosis.

## 1. Introduction

Head and neck squamous cell carcinoma (HNSCC) is the seventh most common cancer worldwide, accounting for 6% of all malignant neoplasms [1], with 660,000 new cases and 325,000 deaths per year [2,3]. The incidence of HNSCC has been increasing in recent years, with a projected increase of 30% by 2030 [1].

Changes in HNSCC epidemiology have been observed, with an increase in the incidence of oropharyngeal tumours due to human papilloma virus (HPV) infection together with a decreasing age at diagnosis [4,5]. This increase means a worsening epidemiological scenario for the medical team, as oropharyngeal and hypopharyngeal carcinomas have a worse prognosis.

The American Joint Committee on Cancer (AJCC) classifies tumour (T) staging systems for head and neck cancers, with the most severe category—T4—divided into two subgroups (T4a and T4b) based on the specific anatomical subsite within the head and neck mucosa. This subdivision aims to highlight the particularly poor prognosis and frequent surgical inoperability associated with T4b tumours. As a result, individuals diagnosed with T4b cancers are typically considered unsuitable for surgical resection, and it is recommended that their treatment prioritises non-surgical options such as chemotherapy, radiotherapy, or a combination of both [6].

HNSCC represents a significant challenge in clinical practice: local advanced head and neck cancers have the characteristic that, while achieving a major resection may be technically feasible, the resulting functional and aesthetic impairments can significantly impact quality of life. However, when the disease spreads extensively and invades vital anatomical structures, it becomes a surgically unresectable tumour. In these cases, despite receiving non-surgical treatments such as chemotherapy, immunotherapy, targeted therapies, and/or radiotherapy, the prognosis is poor and the cause of death is mostly due to tumour progression. Survival rates for patients with advanced HNSCC range from 23 to 43 months, depending on treatment and individual factors [7,8].

A large-cohort study involving a total of 87,844 patients identified that hypopharyngeal tumours had the lowest median overall survival (OS) (4 months), while laryngeal cancer showed the highest median OS (21 months). Staging is critical in providing an accurate prognosis, with stage I having 50% OS after 10 years and stage IVc having a median OS of 3 months [9].

Although there is abundant international literature evaluating survival time in patients with advanced-stage HNSCC, most of these studies include heterogeneous populations, without differentiating between resectable and unresectable cases [5,7,8,9]. Studies of oncologic subpopulations offer the benefit of obtaining specific data on tumour site-specific sequelae, as well as providing more precise information on prognosis and OS for these patients.

This single-cohort follow-up study studies the median OS and the proportion of patients alive after at least 24 months of follow-up in patients with unresectable HNSCC due to invasion of vital structures, which is under-represented in the current literature. Secondarily, subgroups were evaluated according to tumour location and type of treatment to identify clinical characteristics and contribute to the optimisation of therapeutic strategies.

This study does not seek to replace or contradict previous findings, but rather to complement them through a more detailed and useful understanding of the median OS in unresectable HNSCC patients.

## 2. Materials and Methods

This study is an ambispective, single-cohort observational design from 2009 to 2024, which includes 39 patients from the Otorhinolaryngology Department with advanced-stage head and neck cancer with invasion of vital anatomical structures, considered not suitable for surgical treatment. Patients who were considered resectable and opted for radiotherapy or chemoradiotherapy (CRT) as primary treatment were excluded. The study protocol was approved by the institutional review board and informed consent was obtained from all subjects involved in the study.

Patients over 18 years of age diagnosed with surgically unresectable primary tumours and/or cervical adenopathy were included in the study, both with de novo T4b tumours (i.e., those presenting initially with unresectable disease) and with recurrent (Tr) tumours after prior treatment. The cause of death recorded in the medical record was used. Follow-up was 24 months.

All selected patients were diagnosed using the standard protocol. This includes an exhaustive clinical history detailing the complete physical and otorhinolaryngological examination with endoscopic evaluation, biopsy of the tumour, and complementary imaging tests (computed axial tomography (CT), magnetic resonance imaging (MRI) of the regions of interest or total PET CT) to perform a correct staging of the disease. The patients also presented at our hospital’s Multidisciplinary Committee for Head and Neck Tumours, which reached a consensus on the therapeutic approach to be followed on an individual basis. Patients may have been treated with surgery prior to this decision.

The AJCC classification includes 3 repetitive criteria for unresectable cancers across most aerodigestive system sites: (1) encapsulation and invasion of more than 270 degrees of circumferential involvement of the common and internal carotid artery; (2) prevertebral space invasion; and (3) invasion of mediastinal structures. The criteria for unresectability by anatomical region were as follows [9]:

- Oral cavity: This includes invasion of the masticator space, skull base, pterygoid, or internal carotid artery.
- Oropharynx: This includes invasion of the lateral pterygoid muscle, pterygoid, lateral nasopharynx, skull base, or carotid artery.
- Hypopharynx: This includes invasion of the paratracheal fascia, carotid artery, or mediastinal structures.
- Larynx: This includes invasion of the paravertebral space, carotid artery, or mediastinal structures.
- Cancer of unknown primary tumours (CUPs): This includes cases of metastatic cervical adenopathy involving more than 270 degrees of circumferential involvement of the common and internal carotid artery, and invasion of the prevertebral space, mediastinum, or skull base.

### Statistical Analysis

Using the event (patient’s death) variable, a bivariate analysis was conducted to compare proportions across variable levels. Prior to this, the suitability of each variable for parametric testing was assessed. Given that most variables were categorical, either the chi-squared test or Fisher’s exact test was considered. Fisher’s exact test was predominantly used, as many cells in the contingency tables had expected counts below 5; in such cases, the chi-squared test can yield biased or inaccurate estimates.

For time-related data, since it is a numerical variable that does not meet the assumptions required for parametric testing, the Mann–Whitney U test was employed to assess differences in the distribution of time values. However, this type of comparison does not account for censoring in patients who did not experience the event during the study period. Therefore, survival analysis was preferred for time-to-event data.

The survival analysis was carried out using three complementary approaches:

- Kaplan–Meier (KM) analysis: This is a non-parametric method for estimating the survival function over time. It shows the proportion of patients surviving (or remaining event-free) at each time point, generating a survival curve that reflects how survival probability changes over time.
- Log-rank test: This is used to compare survival between two or more groups. It evaluates whether Kaplan–Meier curves differ significantly by comparing observed versus expected events across groups. The test yields a chi-squared statistic and a *p*-value. If *p* < 0.05, there is evidence of a statistically significant difference in survival between groups; if *p* > 0.05, no such difference can be concluded.
- Cox proportional hazards regression model: This is a semi-parametric regression model used to estimate the effect of covariates on the time to event. It provides hazard ratios (HRs), which quantify the relative likelihood of the event occurring in one group compared to another:

HR > 1 → Higher risk of event in that group.

HR < 1 → Lower risk of event in that group.

HR = 1 → No difference in risk.

It also provides confidence intervals and *p*-values to assess statistical significance.

## 3. Results

A total of 39 patients radiologically classified with unresectable HNSCC were included; the mean age was 66.87 years. The majority of patients were male (82.05%, n = 32), while 17.9% (n = 7) were female. The epidemiological characteristics are shown in Table 1.

The causes of death included 15 related to tumour progression, for example, tumour haemorrhage, tumour infection, multi-organ failure, and cardiorespiratory arrest. Three other causes were related to opportunistic diseases or comorbidities such as pneumonia and bronchial aspiration. In many cases, data could not be found due to deaths at home or private centres, where we do not have access to information.

**Figure 1:**
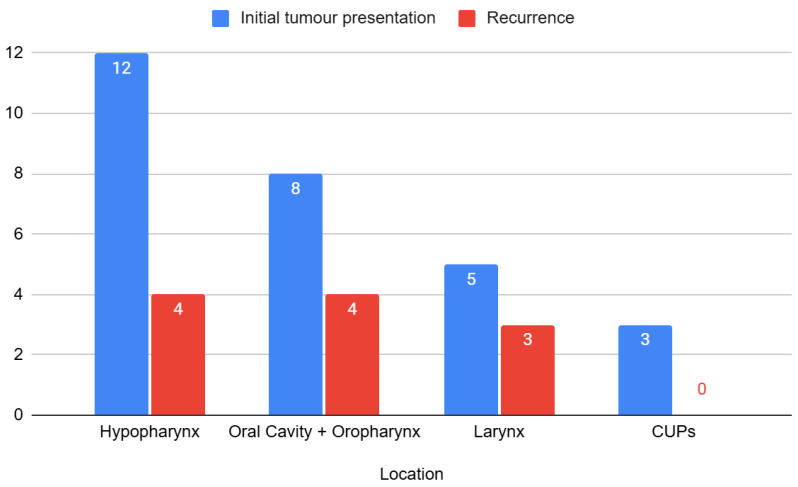
Distribution of neoplasm presentation by anatomical location.

Tumour location was grouped into four groups (Figure 1): hypopharyngeal tumours, representing 41% of the sample (n = 16); oral cavity and oropharyngeal tumours, 30.8% (n = 12); laryngeal tumours, 20.5% (n = 8); and cancer of unknown primary tumours (CUPs), 7.7% (n = 3).

A total of 69.2% of the patients studied had primary unresectable tumours, with the remaining 30.8% being recurrences. Figure 1 shows the relationship between tumour location and type of neoplasm (primary or recurrence).

A total of 20.5% (n = 8) of the patients were positive for HPV, and 79.5% (n = 31) were negative.

In relation to treatment options, chemotherapy, radiotherapy, immunotherapy, and no treatment were considered. In 66.7% of patients two therapeutic modalities were used, with chemoradiotherapy (CRT) being the most used option; in 12.8%, only one therapeutic modality was used, and one patient did not receive any treatment.

Among the chemotherapy treatments, the most commonly used drug was cisplatin, followed by docetaxel, fluorouracil, and carboplatin, with other drugs—such as paclitaxel, taxotere, doxorubicin, and adriamycin—used to a lesser extent. In the immunotherapy group, the patients received nivolumab or pembrolizumab.

By the end of the study, 22 patients (56.4%) had died; the median OS was 16.09 months.

A bivariate analysis was performed to identify associations between clinical and demographic variables and patient death. Statistically significant differences were found for the type of neoplasia (initial/recurrence) (*p*-value = 0.004), showing more overall survival in the case of primary tumours. No significant differences were found for neoplasia location (Table 2).

There were also statistically significant differences for other variables: HPV-positive status presented a greater number of non-deaths, with a *p*-value of 0.013, and the event variable (death/non-death) had a *p*-value of <0.001. Recurrent and HPV-negative tumours had worse survival rates.

The age variable was close to statistical significance, with a *p*-value of 0.050, with the mean age being higher in the deceased group.

The Kaplan–Meier model and log-rank test were used for the survival analysis. The following variables were analysed: location of the tumour, type of tumour (initial/recurrence), and age.

**Figure 2:**
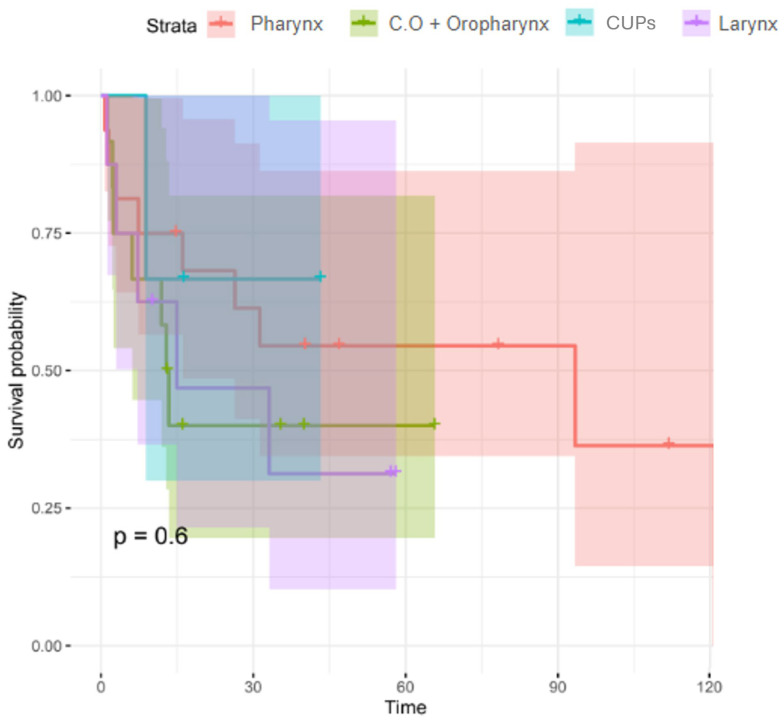
The Kaplan–Meier model and log-rank test used for survival analysis based on tumour localisation.

The median survival rates based on tumour location were 93.39, 13.11, 83.55, and 49.06 months for patients with hypopharyngeal tumours, oral cavity tumours and oropharyngeal tumours, cervical lymph node metastases of unknown primary, and laryngeal tumours, respectively. The log-rank test was used to assess significant differences between groups, giving a *p*-value of approximately 0.6. This indicates that there is insufficient evidence to detect differences in survival in terms of neoplasm location. Figure 2. 

**Figure 3:**
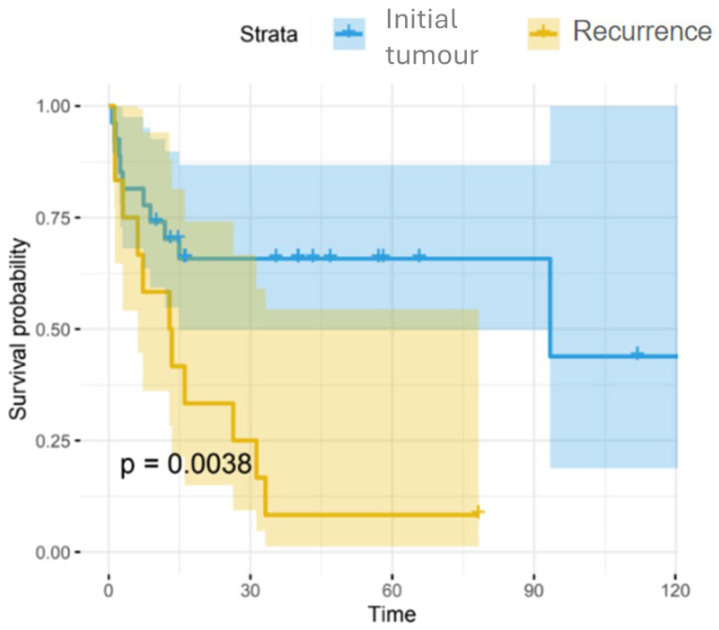
The Kaplan–Meier model and log-rank test used for survival analysis of primary or recurrent tumours.

Significant differences in the type of tumour presentation (initial/recurrence) were observed: the log-rank test gave a *p*-value of approximately 0.0038. In the group of patients with initial tumours, 65.8% of patients were still alive at 14 months; in contrast, for patients in the recurrence group, there was a large decrease in survival at 13–16 months, and only 16.7% remained alive at 30 months, decreasing to 8.3% at 33 months. Figure 3. 

A Cox regression model was used for the type of tumour presentation (initial/recurrence), showing that the recurrence group had a 3.25 times higher risk (hazard ratio = 3.25) of death compared to the primary tumour group, with a statistical significance of *p* < 0.005.

**Figure 4:**
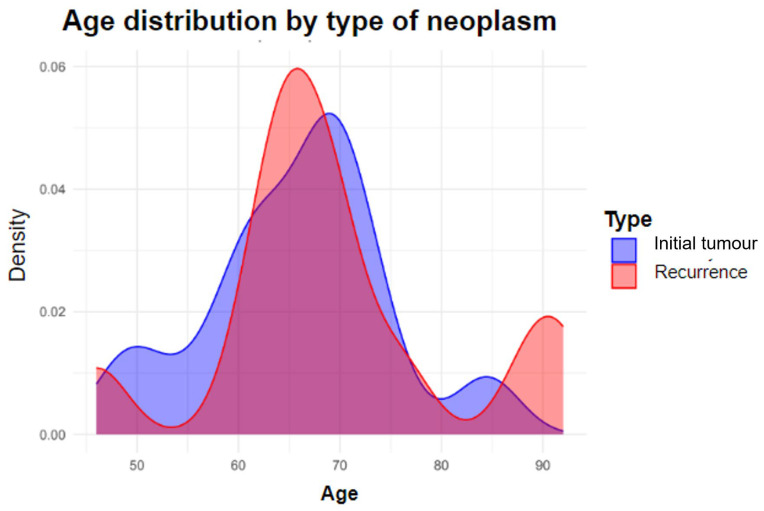
Age distribution by tumour presentation, classified as initial or recurrent.

The age variable was close to statistical significance, with a *p*-value of 0.050, with the mean age being higher in the deceased group. As the oldest patients were in the recurrence group, we evaluated the age distribution per group to see if it was balanced or skewed. It can be seen that the distribution is even, with a *t*-test showing no statistically significant differences. Figure 4.

Taking into account the difference in prognosis in recurrent tumours, the analysis was deepened by separating the groups into tumours of initial or recurrent presentation. In addition, CUP patients were excluded due to their distinct biological behaviour, diagnostic uncertainty, and the potential for significant heterogeneity in treatment response, which may confound survival outcomes in a cohort otherwise defined by anatomical site and known primary tumour characteristics. From the total of 36 patients, 12 patients were in the recurrence group and 24 in the initial tumour group.

In the recurrence group, the OS was 13.11 months, and the mean age was 69.25 years. Four patients had tumours located in the larynx, four in the oral cavity + oropharynx, and four in the hypopharynx. No HPV-positive cases were observed in this group. Eleven (91.7%) cases in this group died during follow-up. Table 3.

In the group with initial tumours, the OS was 25.72 months, and mean age was 66.21 years. Twelve patients had a tumour in the hypopharynx, eight in the oral cavity, and four in the larynx. Eight patients were HPV-positive (33.3%), and fourteen survived during follow-up (58.3%) Table 4. Apart from time (OS), there are no other significant variables.

With regard to the therapies received, in the recurrence group (12 patients), 7 patients received immunotherapy, and bicombination therapy was used in 54.5% of patients. In the initial tumour group (26 patients), 9 patients received immunotherapy (37.5%), and 75% received bicombination therapy, higher than in the recurrence group. In the bivariate analysis of both groups, no variable was significantly associated with prognosis.

## 4. Discussion

This study focuses on cancer resectability, as a critical determinant of patient prognosis. Imaging plays a pivotal role in assessing the resectability of head and neck cancers; the determination of unresectability should, in part, rely on the most advanced and accurate imaging modalities available.

The literature highlights that certain anatomical regions pose particular challenges in evaluating tumour invasion, often requiring a multimodal imaging approach and expert assessment by an experienced multidisciplinary team. MRI has demonstrated utility in detecting invasion of the carotid artery and prevertebral space [6].

Assessing recurrence or residual disease in patients previously treated with CRT for advanced head and neck cancer remains complex. These individuals are frequently ineligible for additional curative doses of radiation or chemotherapy. In this context, PET/CT fusion imaging has proven especially valuable. Both surgery and CRT induce anatomical changes that can limit the diagnostic accuracy of conventional imaging modalities such as CT and MRI. Early identification of tumour recurrence or persistence is essential to enhance local disease control and improve survival outcomes in patients undergoing salvage surgery [6].

The results of this ambispective single-cohort follow-up study confirm that patients with unresectable head and neck cancer due to the invasion of vital anatomical structures have a poor prognosis, with a significantly reduced median survival compared to those with resectable disease. The median OS in this study was 16.09 months, 13.11 months in the recurrence group and 25.72 months in the group with an initial tumour—compared with 22–43 months in previous studies [7,8]. A systematic review on palliative radiotherapy in patients with locally advanced non-metastatic disease reported a median survival of between 3.3 and 17 months [10].

Several prognostic factors have been identified in advanced HNSCC, including the TNM stage, the primary tumour size, and comorbidities [6,7,8]. The TNM stage is the main prognostic factor for treatment outcome; in stage IV HNSCC, long-term survival is rare, and the presence and the number of metastases is a significant predictor for mortality [7,8]. 

Other influential factors in prognosis of advanced-stage cancer patients include the primary tumour volume, which has been identified as a predictor of disease control and survival in patients with advanced HNSCC treated with CRT [11]. 

The patient’s overall clinical condition is another key determinant of disease progression. Comorbidities in HNSCC patients are mainly related to cardiopulmonary, vascular, hepatic, and metabolic diseases; they elevate the risks associated with anaesthesia, surgery, and postoperative complications. The presence of significant medical comorbidities not only renders the patient intolerant of extensive surgical resections but also limits the efficacy of radiotherapy and chemotherapy, which are the mainstay of treatment in unresectable cases. Furthermore, comorbidities at the time of diagnosis of HNSCC have been associated with therapeutic delay, increased risk of 30-day mortality after treatment, and lower survival, with comorbid patients reported to be more likely to die prematurely due to concomitant diseases compared to those without comorbidities [12,13]. 

Baseline comorbidity must be recognised as an independent prognostic factor influencing post-treatment mortality, as well as overall and relative survival, in patients with HNSCC. Clinical decision-making tools should systematically incorporate comorbidity assessments to guide individualised treatment strategies. Given that patients with significant comorbid conditions are frequently excluded from clinical trials, the applicability of trial-derived guidelines to real-world populations is often limited. We strongly advocate for the inclusion of patients with relevant comorbidities in future clinical trials to better reflect everyday clinical practice [12].

Although geriatric assessment tools provide some guidance for managing elderly patients, further research is urgently needed to address supportive care needs in other high-risk subgroups. The emphasis must shift toward a patient-centred approach in HNSCC management, moving beyond the traditional disease-centred paradigm, to ensure care is tailored to the individual’s overall health context [12].

Our results confirm that tumour recurrence is another determining factor of prognosis of patients with unresectable HNSCC. Recurrent HNSCC disease is associated with high rates of incurability and poorer survival than primary cancers. Poor prognosis of tumour recurrence is well known in the literature in patients with advanced-stage tumours in general. According to Williamson, from a cohort of 1488 patients, the OS at two years was 41.1%. Even in recurrences, surgical cases had better OS than patients with non-surgical treatments; compared to primary cancers, the recurrent HNSCC group showed a more advanced T stage (*p* < 0.001) and distant metastasis (*p* < 0.001), received less curative treatment (*p* < 0.001), and had worse survival outcomes (all *p* < 0.001) [14]. As expected, our unresectable patients also showed worse survival.

Consistent with our findings, HPV-negative status has emerged as a strong and independent prognostic factor for both progression-free survival and disease-free survival among patients with oropharyngeal cancer [15,16,17,18]. HPV-positive patients experience significantly lower disease-specific mortality and are less likely to experience progression or recurrence, which has important treatment implications [19]. 

High-risk HPV—especially type 16—is a major risk factor for oropharyngeal cancer and is thought to be sexually transmitted through orogenital contact [20,21,22]. Having >4 oral sex partners has been shown to significantly increase the risk of oropharyngeal cancer [23]. HPV has been variably associated with oropharyngeal cancer worldwide, with a notable association observed in over 50% of cases in the United Kingdom [24,25,26]. Smoking has also been shown to interact with HPV and increase the risk [27].

Interestingly, individuals with HPV-negative oropharyngeal tumours are more likely to be heavy smokers and face a greater risk of death with each additional pack-year, compared to HPV-positive cases [15]. HPV-positive cases exhibit a nearly 60% reduction in mortality risk after adjustment for prognostic factors such as age, ethnicity, staging, smoking status, and treatment regimen [15]. This may be because individuals with HPV-positive disease tend to be slightly younger, have fewer comorbidities, or possess enhanced antitumour immunity. It has also been suggested that HPV-positive tumours harbour fewer genetic mutations or may be more radiosensitive (with an intact apoptotic response), which correlates with improved overall response to radiotherapy [28].

The current standard treatment for advanced head and neck cancer involves combining radiation therapy with chemotherapy. A meta-analysis showed that none of the alternative treatments demonstrated significant advantages over the standard concurrent CRT; some studies found that adding PD-1/PD-L1 inhibitors to standard therapy does not enhance 1- or 2-year survival for patients with local advanced HNSCC [29]. Nevertheless, there is potential for improved outcomes when targeted therapy or induction chemotherapy is combined with concurrent CRT [30]. It is not the final aim of this study to analyse the characteristics of each therapeutic modality; we only mention that in our study CRT was the most commonly used option in 66.7% of patients. A systematic review supports the use of a variety of therapeutic combinations for local advanced HNSCC patients ineligible for cisplatin [31].

In our study, there was one patient who did not receive any treatment and survived for 22 days. There is evidence in the literature that the median survival for untreated patients is 12 months (95% CI: 11–13 months), compared to 100 months (95% CI: 98–103 months) for treated patients. Advanced age, unmarried status, and insurance status were strongly associated with being untreated [9,32,33].

In our study, we had 7.7% of unresectable patients in cases of cancer of unknown primary tumours (CUPs). In these cases, the search for the primary tumour must always be carried out clinically and radiologically. In many cases, it is only discovered by means of deep biopsies in the operating theatre. In addition to the classical locations such as the tonsils, base of the tongue, larynx, and hypopharynx, the posterior wall of the oropharynx should be carefully investigated. Identification of the primary tumour on clinical examination and imaging studies reduces the need for diagnostic surgery [34]. However, as presented in our study, rapid progression of tumour growth makes cervical adenopathy unresectable. In a previous study, 965 patients treated as cases of CUPs underwent survival analysis, stratified by neck dissection and/or radiotherapy to the ipsilateral neck and by HPV status; HPV-negative status was associated with worse OS [35].

This study has some limitations, one of which is that the small sample size (n = 39 with further subdivision into smaller groups) significantly restricts the statistical robustness and interpretive strength of subgroup analyses, especially regarding differences between initial and recurrent tumours. Another limitation is that detailed retrospective data on prior treatments, especially in recurrent cases, was limited or unavailable, thus restricting interpretation of treatment-related outcomes. Our findings must be interpreted as preliminary, hypothesis-generating, and not definitive, considering the inability to provide a comprehensive comparative analysis with the existing literature due to heterogeneity of previous studies and the small sample size in the current study.

The study population is rare but clinically relevant, as knowing and adjusting life expectancy for patients may help with the uncertainty that a cancer diagnosis produces. The homogeneity of the cohort provides valuable real-world data on treatment outcomes in patients not eligible for surgery.

This study provides informed data to advise patients on prognosis, which is essential for developing an appropriate treatment plan and managing patients’ expectations. To date, there is a lack of robust data on the outcomes of patients with unresectable tumours. To our knowledge, few studies have exclusively addressed the survival outcomes of patients with unresectable T4b and recurrent head and neck cancers as distinct entities. Our findings suggest differing prognostic trajectories between these subgroups, warranting further investigation. 

## 5. Conclusions

In conclusion, invasion of critical anatomical structures such as the skull base, internal carotid artery, and prevertebral space renders tumours unresectable and is therefore associated with a marked decrease in survival. The OS in these cases was approximately 16 months. This study provided valuable data on patients with unresectable HNSCC, such as the possible influence of tumour recurrence and HPV-negative status as important indicators of poor prognosis.

## Figures and Tables

**Table 1:** Epidemiological characteristics.

		Overall
n		39
Sex	Male	32 (82.1%)
	Female	7 (17.9%)
Tumour location	Hypopharynx	16 (41.0%)
	Oral cavity + oropharynx	12 (30.8%)
	CUPs	3 (7.7%)
	Larynx	8 (20.5%)
Initial tumour presentation or recurrence	Initial	27 (69.2%)
	Recurrence	12 (30.8%)
HPV-positive	Yes	8 (20.5%)
	No	31 (79.5%)
Radiotherapy	Yes	31 (79.5%)
	No	8 (20.5%)
Chemotherapy	Yes	29 (74.4%)
	No	10 (25.6%)
Immunotherapy	No	35 (89.7%)
	Yes	4 (10.3%)
Event (death)	No	17 (43.6%)
	Yes	22 (56.4%)
Cause of death	Tumour progression	15 (38.5%)
	Opportunistic diseases/comorbidities	3 (7.7%)
	Not Applicable	21 (53.8%)
Time (median [iqr])		16.05 [7.30, 40.20]
Age (mean (sd))		66.87 (10.08)

**Table 2:** Bivariate analysis between clinical and demographic variables and patient death.

		Overall	No	Yes	*p*
n (%)		39	17	22	
Sex	Male	32 (82.1)	16 (94.1)	16 (72.7)	0
	Female	7 (17.9)	1 (5.9)	6 (27.3)	
Location of neoplasm	Hypopharynx	16 (41)	7 (41.2)	9 (41)	0.864
	Oral cavity + Oropharynx	12 (30.8)	5 (29.4)	7 (31.8)	
	CUPs	3 (7.7)	2 (11.8)	1 (4.5)	
	Larynx	8 (20.5)	3 (17.6)	5 (22.7)	
Tumour presentation	Initial	27 (69.2)	16 (94.1)	11 (50)	0.004
	Recurrence	12 (30.8)	1 (5.9)	11 (50)	
HPV status	Yes	8 (20.5)	7 (41.2)	1 (4.5)	0.013
	No	31 (79.5)	10 (58.8)	21 (95.5)	
Event of death	Yes	22 (56.4)	0 (0)	22 (100)	<0.001
	No	17 (43.6)	17 (100)	0 (0)	
Cause of death	Tumour progression	15 (83.3)	0 (0)	15 (83.3)	1
	Opportunistic or concomitant diseases	3 (16.7)	0 (nan)	3 (16.7)	
Time (median [iqr])		16.05 [7.30, 40.20]	40.20 [16.28, 57.11]	8.09 [2.57, 15.80]	<0.001
Age (mean (sd))		66.87 (10.08)	63.29 (9.41)	69.64 (9.91)	0.05

**Table 3:** Bivariate analysis based on death with recurrences. We observe that we do not have any significant variables. In fact, the *p*-values are far from significant.

		Overall	No	Yes	*p*
n		12	1	11	
Sex	Male	7 (58.3)	1 (100.0)	6 (54.5)	1.0001
	Female	5 (41.7)	0 (0.0)	5 (45.5)	
HPV-positive	No	12 (100.0)	1 (100.0)	11 (100.0)	NA
Event of death	No	1 (8.3)	1 (100.0)	0 (0.0)	0.083
	Yes	11 (91.7)	0 (0.0)	11 (100.0)	
Time (median [IQR])		13.11 [5.36, 27.57]	78.26 [78.26, 78.26]	12.86 [4.57, 21.22]	0.111
Age (mean (SD)		69.25 (12.24)	65.00 (NA)	69.64 (12.76)	NA

**Table 4:** Bivariate analysis based on death with initial tumours.

		Overall	No	Yes	*p*
n		24	14	10	
Sex (%)	Male	22 (91.7)	13 (92.9)	9 (90.0)	1.000
	Female	2 (8.3)	1 (7.1)	1 (10.0)	
HPV-positive (%)	Yes	8 (33.3)	7 (50.0)	1 (10.0)	0.079
	No	16 (66.7)	7 (50.0)	9 (90.0)	
Event of death (%)	No	14 (58.3)	14 (100.0)	0 (0.0)	<0.001
	Yes	10 (41.7)	0 (0.0)	10 (100.0)	
Time (median [IQR])		25.72 [9.39, 49.46]	40.20 [20.89, 54.56]	5.16 [2.29, 14.18]	0.022
Age (mean (SD)		66.21 (9.42)	63.14 (10.27)	70.50 (6.26)	0.057

## Data Availability

The original contributions presented in this study are included in the article. Further inquiries can be directed to the corresponding author.
